# Human indoor climate preferences approximate specific geographies

**DOI:** 10.1098/rsos.180695

**Published:** 2019-03-20

**Authors:** Michael G. Just, Lauren M. Nichols, Robert R. Dunn

**Affiliations:** 1 Department of Entomology and Plant Pathology, North Carolina State University, Raleigh, NC, USA; 2 Department of Applied Ecology, North Carolina State University, Raleigh, NC, USA; 3 Center for Macroecology, Evolution and Climate, University of Copenhagen, Copenhagen, Denmark

**Keywords:** climate dissimilarity, human niche construction, human associates, thermal comfort, indoor biome

## Abstract

Human engineering of the outdoors led to the development of the indoor niche, including home construction. However, it is unlikely that domicile construction mechanics are under direct selection for humans. Nonetheless, our preferences within indoor environments are, or once were, consequential to our fitness. The research of human homes does not usually consider human evolution, and, therefore, we are without previous predictions about indoor climate preference. We worked with citizen scientists to collect indoor climate data from homes (*n* = 37) across the USA. We then compared these data to recent global terrestrial climate data (0.5° grid cells, *n* = 67 420) using a climate dissimilarity index. We also compared some climate-related physiological parameters (e.g. thermoneutral zone (TNZ)) between humans and a selection of non-human primates. On average, our study homes were most similar in climate to the outdoor conditions of west central Kenya. We found that the indoor climates of our study homes largely matched the TNZ of humans and other primates. Overall, we identified the geographical distribution of the global outdoor climate that is most similar to the interiors of our study homes and summarized study home indoor climate preferences.

## 1. Introduction

Climate plays an important role in the life history of most organisms, and the influence of climate on the ecology, evolution and distribution of organisms has been the subject of many thousands of studies. Similarly, outdoor climates themselves have been the subject of a rich body of work, both in terms of current climate, projected future climate and modelled or measured historic climates. Yet, somehow, the relationship between humans and climate, particularly the climate in the ecological realm we spend the most time in, our homes, remains poorly studied, particularly with regard to the ecology and evolution of humans and the many thousands of species that live alongside us [1,2].

Dawkins coined the term *extended phenotype* to describe the extent to which an organism's genes encode not only its body and behaviour but also the ways in which that organism might manipulate the environment [3]. The termite's nest is part of its extended phenotype [4] and is mediated both by genes associated with behaviour and the rules those genes influence, just as the warren of a mouse is part of its [5]. Recent work has even begun to understand the individual genes associated with deer mice (*Peromyscus* spp.) and when they build one type of warren relative to another [6]. But what about humans? It would be difficult to convincingly argue that the behaviours leading to the construction of human houses are under direct selection. Many humans (the authors of this paper included) could not build a modern house if their life depended on it, yet we persist. However, the issue may be more subtle than it at first seems; human preferences influence human houses. Our houses are built to reflect both comfortable temperatures and levels of humidity [7,8]. If our house is too hot or cold, we modify it in such a way as to produce more heat and vice versa [9]. However, for thousands of years before air conditioning, we also modified conditions through construction or placement of homes that buffered outdoor climates with passive measures such as sun shading, thermal mass and ceiling architecture, to both to make them liveable and to make them comfortable [10,11].

For ectotherms, a large body of the literature considers how individual organisms alter their climate [12]. Species seek favoured climates or employ body postures that alter the temperature to which they are exposed [13–16]. In social insects, some species even alter the climate around them, and particularly their brood, whether through collective behaviours (e.g. honey bees [17]) or through the constructions the behaviours create (e.g. nests [18]). Similar phenomena are reported for mammals, but often anecdotally, especially for primates including humans [19]. The relationship of humans with climate is complex [20,21]. We thermoregulate [22], acclimate [23], and, over time, we have even adapted in as much as individual human lineages appear to demonstrate physiological and anatomical differences associated with their historic climates [24]. Yet, the defining way in which we have responded to outdoor climate, since the advent clothing, no less than 20 000 years ago, is to modify the climate we are exposed to in order to maximize thermal comfort [25].

A rich literature considers the many proximate factors that influence thermal comfort. Thermal comfort can be influenced by culture [11,26], by wind speed and humidity [27–30] and mean radiant temperature [31,32]. This literature suggests that the many ways in which the climate people prefer for their homes might be modulated and why. But what these do not change is the reality that thermal comfort itself, evolved.

What do we favour about these indoor climatic conditions? Are they similar to the climate of our ancestors? Which (outdoor) climate are we attempting to reconstruct when we turn the heat up or down? These questions seem to have been given little consideration, perhaps for two reasons. First, there is a paucity of reported indoor climate data across seasons for occupied homes, which would allow direct comparison with outdoor climates except where specific house types are being compared (e.g. traditional versus modern homes [26,27]). Second, the people who study indoor environmental quality (e.g. homes and their interior climates) do so in the context of creating interior spaces that promote comfort and productivity rather than in an ecological or evolutionary context [33,34]. Understanding the climates humans construct in light of human ecology and evolution has relevance not only to understanding why we build homes the way we do (and how we might make more reasoned decisions in the future), but also the climate that we create for other organisms indoors. The indoor biome is one of the most rapidly growing biomes on Earth [35], yet its climatic features have not been well characterized with regards to species ecology, nor have they been compared to other, outdoor climates. Such a comparison is necessary in order to understand which climates we have replicated indoors and which species might be most predisposed, in terms of climate, to live with us in the future, whether wanted or unwanted. As many as several hundred thousand species have been found living in homes [1,2], and the question of the climate that these species inhabit is relevant to the basic biology of a broad swathe of life.

Here, we worked with citizen scientists to record the climate within homes across the USA. We first characterize the indoor climates of these homes, then we compare these indoor climates with what is known about the climatic tolerances of non-human primates, and finally, we identify specific geographies from across the globe whose climate is most similar to the observed indoor climates. In considering which (global) outdoor climates these North American homes are most similar to, we argue that there are two consequences of the conditions that we prefer in our homes. First, the climates we prefer have strong effects on global energy usage and how that usage varies geographically. Second, and perhaps less obviously, in constructing our homes and modulating their climate as an extension of our phenotype (and to some extent culture) we might also recreate specific climates for other organisms, favouring the subset of species that prefer the same climates as we do [35].

## 2. Material and methods

### 2.1. Climate datasets

With the assistance of citizen scientists, we collected indoor and outdoor climate data from homes from each state of the USA and Washington, DC using a temperature (°C) and relative humidity (%) data logger (iButton model DS1923-F5, Maxim Integrated Products, Inc., Sunnyvale, CA, USA) that is commonly used in ecological studies [36]. For indoor climate measurements, participants were instructed to place the data logger on a surface with a low risk of physical disturbance, and away from any air vents, windows or direct sunlight (e.g. shelf, bookcase). Participants were asked to place the outdoor climate logger in a location that was disturbance free and shaded. The data collection period was February 2013–April 2014, and temperature and humidity were recorded once per hour. During initial data processing, prior to analysis, we removed homes that did not have records from summer, winter and, at least, spring or autumn; 37 homes were retained (additional information on study homes available as electronic supplementary material, table S1). To align the indoor air moisture variable with that of the global outdoor data, we calculated vapour pressure (hPa) from indoor temperature and relative humidity observations using the August–Roche–Magnus equation [37]. Home climate data were converted to monthly averages prior to analyses. We examined the relationship between indoor and outdoor home temperatures with linear regression, fitting regressions for both vapour pressure and temperature by season. All analyses were performed in R [38] (version 3.3.2; http://www.R-project.org). This research was approved by the NC State University IRB review board under IRB Protocol 2177. We received written consent from all participants.

Global, outdoor climate data were acquired from the University of East Anglia Climatic Research Unit's Time-Series Version 3.21 High Resolution Gridded Data [39] (CRU TS3.21; http://catalogue.ceda.ac.uk). This dataset is constructed from monthly observations from terrestrial meteorological stations from across the globe. Station anomalies are interpolated to 0.5° grid cells (*n* = 67 420 terrestrial cells excluding Antarctica) and combined with an existing climatology [40] to derive absolute monthly values. We used the 2012 CRU TS3.21 monthly air temperature (°C) and vapour pressure (hPa) data for our analyses.

### 2.2. Climate dissimilarity

We calculated the dissimilarity between North American indoor and global outdoor climates, using the climatic parameters air temperature (°C) and vapour pressure (hPa), to determine if indoor climates approximated outdoor climates of specific geographies. For our dissimilarity analyses, we used six climate variables: minimum mean air temperature and mean vapour pressure for winter, mean air temperature and mean vapour pressure for spring/autumn, and maximum air temperature and mean vapour pressure for summer. Seasons were defined as follows for the Northern and Southern Hemispheres, respectively: December–February (winter/summer), March–May (spring/autumn), June–August (summer/winter), September–November (autumn/spring). Spring and autumn were analysed as one season, averaging spring and autumn values as needed. Air temperature and air moisture are often-used climatic variables when considering indoor climate and human thermal comfort [29,41]. These parameters have also been used in studies of climate analogues [42].

We used a standardized Euclidian distance to compute a climate dissimilarity index [42,43] between each home and global grid cell (equation (2.1)), using the climate variables described above.

2.1 $$C_{ij}=\;\sqrt{\overset{6}{\underset{k=1}{\sum }}\frac{{\left(g_{kj}-\;h_{ki}\right)}^{2}}{S_{ki}^{2}}},$$

where *C* is the climate dissimilarity index between each indoor *i* and outdoor *j* location. Where *k* is the climate variable (*n* = 6), *h* is the mean of the indoor climate variable *k* at *i*, *g* is the mean outdoor climate variable *k* at *j*, and $S_{ki}^{2}$ is the standard deviation of the indoor climate variable. Climate dissimilarity indices are a common tool used to compare climates separated by space and/or time and to find the climate that is most or least similar to a focal climate [42,44–46]. We also calculated the root mean square errors for temperature and vapour pressure between each home and global grid cell, and the methods and results of these analyses can be found in the electronic supplementary material, appendix A.

## 3. Results

### 3.1. Indoor climates

**Figure 1.**
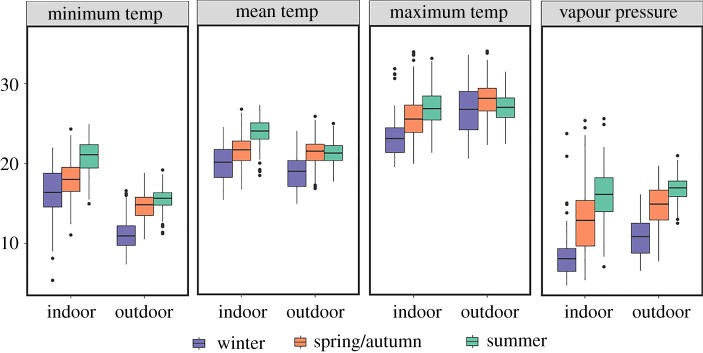
Boxplots for the climatic variables air temperature (°C) and vapour pressure (hPa) by season (spring and autumn are averaged; winter = purple, spring/autumn = orange, summer = green) and location. Minimum temp is the mean minimum air temperature, mean temp is mean air temperature, maximum temp is the mean maximum air temperature and vapour pressure is the mean vapour pressure. The indoor climate is from our study homes and outdoor climate values are from the 100 grid cells that are the most climatically similar to the mean home indoor climate. The box plots display data range, quartiles and median with dots as outliers. Figure was generated with R (version 3.3.2; http://www.R-project.org) package *ggplot2* (version 2.2.0; http://CRAN.R-project.org/package=ggplot2).

The mean maximum temperature in the summer for the 37 homes ranged from 22.22 to 34.63°C, with a mean of 27.27 ± 0.46°C (standard error of the mean); mean vapour pressure ranged from 10.22 to 25.28 hPa with a mean of 16.15 ± 0.46 hPa (figure 1). The mean minimum temperature in winter ranged from 8.38 to 22°C, with a mean of 16.44 ± 0.52°C, and mean vapour pressure ranged from 4.98 to 22.33, with a mean of 8.75 ± 0.54 hPa. The mean temperature of spring/autumn ranged from 17.52 to 25.37°C, with a mean of 21.51 ± 0.28°C, and mean vapour pressure ranged from 8.26 to 23.59 hPa, with a mean of 12.82 ± 0.46 hPa.

Outdoor air temperature was a significant predictor of indoor home temperature by season, but the strength of these associations was modest (table 1). The relationship between outdoor and indoor temperature was especially weak in winter (*t* = 2.88, adjusted *R*^2^ = 0.04, *p* < 0.001). Associations of outdoor and indoor home vapour pressure were generally stronger than the same comparisons for temperature (table 1). The weakest relationship between outdoor and indoor home vapour pressure was found in summer (*t* = 7.69, adjusted *R*^2^ = 0.30, *p* < 0.001).

**Table 1.** Results of linear models evaluating indoor home climate (temperature, vapour pressure) by outdoor home climate.

season	d.f.	variable	estimate (s.e.)	*t* value	adj. *R*^2^
winter	159	temperature (°C)	0.063 (0.02)	2.88**	0.044
		vapour pressure (hPa)	0.757 (0.03)	23.41***	0.774
spring/autumn	218	temperature (°C)	0.246 (0.02)	14.13***	0.476
		vapour pressure (hPa)	5.862 (0.33)	23.44***	0.715
summer	136	temperature (°C)	0.245 (0.03)	8.23***	0.328
		vapour pressure (hPa)	0.351 (0.05)	7.69***	0.298

Significance levels **p < 0.01, ***p < 0.001.

### 3.2. Most similar indoor and outdoor climates

**Figure 2.**
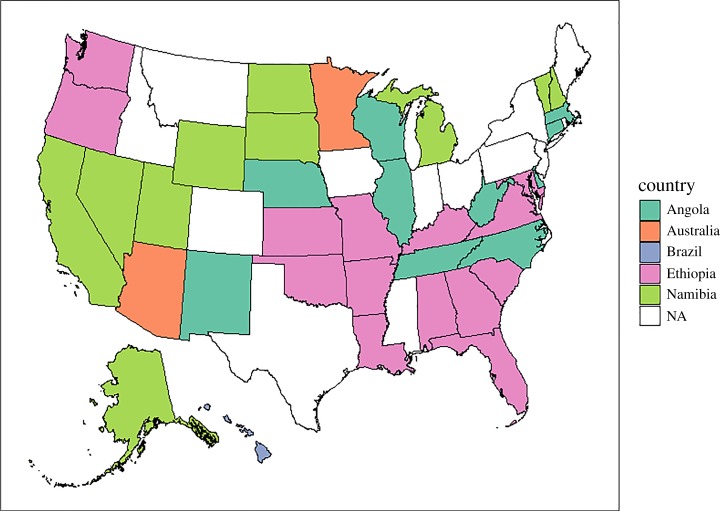
Map of the USA (not to scale). Each state represents one study home (*n* = 37). State fill colour represents the country (Angola, aquamarine; Australia, salmon; Brazil, light purple; Ethiopia, magenta; Namibia, green) that was identified as most frequent from a subset of the 100 global grid cells with most similar climate to each study home. States not included in the analysis are shown in white. Map was generated with R (version 3.3.2; http://www.R-project.org) packages *ggplot2* (http://CRAN.R-project.org/package=ggplot2), *mapproj* (version 1.2-4; https://cran.r-project.org/package=mapproj), *rgdal* (version 1.2-7; https://cran.r-project.org/package=rgdal) and *sp* (version 1.2-4; https://cran.r-project.org/package=sp). State boundaries (5 m resolution) were obtained from the US Census Bureau (https://www.census.gov/geo/maps-data/data/cbf/cbf_state.html).

We identified the outdoor location(s) with the most similar climate for each of our study homes (table 2). The indoor climate from the Oregon home, for example, had the smallest observed *C* and was a close match (*C* = 0.3812) with a grid cell in Kenya (0.25° N, 35.25° E). By contrast, the indoor climate for the Missouri home had the greatest minimum *C* (3.765) for its most similar outdoor climate (1.75° N, 35.25° E) which was also located within Kenya. To generalize the climate similarities, we also considered the 100 most similar outdoor climates for each home. The Hawaii home had the lowest mean *C* (0.900 ± 0.014) and these global grid cell centres that were most often located in Brazil (figure 2) and the Missouri home also had the greatest minimum mean *C* (4.120 ± 0.017), and the locations of these global grids most often occurred in Ethiopia.

**Table 2.** Results of climate dissimilarity analysis between the indoor climate of a North American home (*n* = 37) and 67 420 global terrestrial grid cells. *C*_nearest_ is the minimum value of the climate dissimilarity index (*C*) for that state. The country where the centre (latitude and longitude) of the grid cell is located is listed as the nearest country. *C*_Top 100_ is the mean minimum value of *C* (standard error) for the 100 most climatically similar global grid cells for that state, the corresponding country represents the most frequently observed country from the 100 most climatically grid cells.

state	*C*_nearest_	country (nearest)	latitude	longitude	*C*_Top 100_	country (top 100)
Alabama	2.557	Kenya	1.75	35.25	3.181 (0.022)	Ethiopia
Alaska	1.273	Namibia	13.75	−19.75	1.879 (0.023)	Namibia
Arizona	2.146	Namibia	−22.75	15.75	2.547 (0.012)	Australia
Arkansas	1.221	Ethiopia	7.75	35.25	1.79 (0.019)	Ethiopia
California	1.263	Namibia	−21.25	14.75	1.868 (0.019)	Namibia
Connecticut	2.197	Namibia	−22.25	15.25	2.625 (0.018)	Angola
Delaware	0.770	Angola	−13.75	16.25	1.272 (0.025)	Angola
Florida	1.705	Ethiopia	3.75	38.75	2.339 (0.028)	Ethiopia
Georgia	1.755	Ethiopia	9.75	35.25	2.317 (0.017)	Ethiopia
Hawaii	0.561	Brazil	−11.25	−38.25	0.9 (0.014)	Brazil
Illinois	2.648	Namibia	−21.75	15.25	3.249 (0.017)	Angola
Kansas	2.087	Kenya	0.75	35.75	2.64 (0.016)	Ethiopia
Kentucky	2.163	Kenya	1.75	35.25	2.62 (0.017)	Ethiopia
Louisiana	1.258	Kenya	−1.25	38.25	1.658 (0.015)	Ethiopia
Maryland	3.243	Ethiopia	9.75	35.25	3.759 (0.019)	Ethiopia
Massachusetts	0.736	Angola	−15.75	14.75	1.329 (0.023)	Angola
Michigan	2.188	Namibia	−22.75	15.25	2.742 (0.014)	Namibia
Minnesota	3.079	Bermuda	32.25	−64.75	3.562 (0.011)	Australia
Missouri	3.580	Ethiopia	12.75	37.25	4.12 (0.017)	Ethiopia
Nebraska	1.745	Angola	−10.75	22.25	2.347 (0.019)	Angola
Nevada	3.210	Namibia	−21.75	15.75	3.945 (0.027)	Namibia
New Hampshire	1.898	Namibia	−21.25	14.75	2.457 (0.018)	Namibia
New Mexico	2.487	Ethiopia	12.75	37.25	3.1 (0.019)	Angola
North Carolina	1.519	Ethiopia	10.75	35.75	1.792 (0.009)	Angola
North Dakota	2.970	Namibia	−21.75	15.25	3.613 (0.018)	Namibia
Oklahoma	2.820	Kenya	1.75	35.25	3.387 (0.019)	Ethiopia
Oregon	0.387	Kenya	0.25	35.25	1.109 (0.023)	Ethiopia
South Carolina	1.967	Ethiopia	4.25	39.25	2.511 (0.021)	Ethiopia
South Dakota	2.721	Namibia	−21.25	14.75	3.267 (0.018)	Namibia
Tennessee	1.272	Namibia	−22.25	15.25	2.08 (0.021)	Angola
Utah	1.982	Namibia	−21.25	14.75	2.565 (0.018)	Namibia
Vermont	1.719	Mexico	25.25	−106.75	2.078 (0.012)	Namibia
Virginia	1.658	Ethiopia	9.75	35.25	2.328 (0.018)	Ethiopia
Washington	1.870	Kenya	1.25	35.75	2.498 (0.02)	Ethiopia
West Virginia	1.561	Ethiopia	10.75	35.75	1.941 (0.012)	Angola
Wisconsin	1.997	Namibia	−21.75	14.75	2.538 (0.016)	Angola
Wyoming	3.433	Namibia	−22.75	15.75	4.119 (0.023)	Namibia

Considering all global cells (*n* = 67 420), the location with the least similar climate to the mean North American indoor climate was located within northern Greenland (79.75° N, 39.25° W; *C* = 39.874). In other words, to achieve the indoor conditions found in North America, someone in Greenland would have to alter indoor conditions relative to outdoor conditions more than anywhere else on Earth. Conversely, the location with the most similar climate was located in west central Kenya (1.25° N, 35.75° E; *C* = 2.938). In west central Kenya, outdoor conditions are essentially the same as the mean conditions created inside homes in North America.

**Figure 3.**
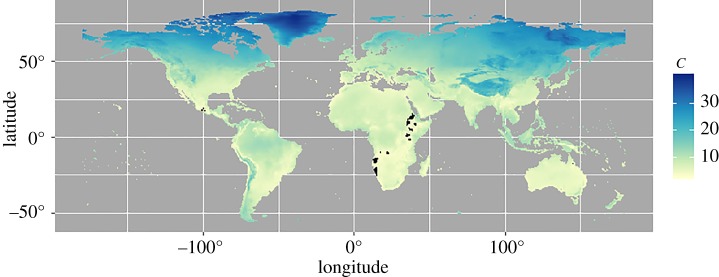
Map depicting the climate dissimilarity index (*C*) between the mean indoor climate of the North American homes (*n* = 37; 2013–2014) and the outdoor climate of terrestrial 0.5° global grid cells (*n* = 67 420; 2012). Dissimilarity increases as *C* increases (yellow to blue). Cells depicted in black are those grid cells with the climatic conditions most similar to the average North American home in terms of temperature and humidity (*n* = 100). Map was generated with R (version 3.3.2; http://www.R-project.org) packages *ggplot2* (version 2.2.0; http://CRAN.R-project.org/package=ggplot2), *rgdal* (version 1.2-7; https://cran.r-project.org/package=rgdal), *sp* (version 1.2-4; https://cran.r-project.org/package=sp), and *rworldmap* (version 1.3-4; https://cran.r-project.org/package=rworldmap), which uses Natural Earth data (version 1.4.0; http://www.naturalearthdata.com) for country borders.

We were interested in identifying potential global, outdoor locations from which the species associated with North American homes might be most expected to have come. To this end, we used the overall mean climatic dissimilarity metric (*C*) from our study homes to identify the 100 (of 67 420) most similar global grid cells (figure 3). The value distributions of the climate variables used in the climate dissimilarity index can be viewed in figure 1.

## 4. Discussion

Here, we present data on observed indoor climate from homes across the North America (figure 1). Indoor environments are important for humans; the average person in the USA spends, for example, less than 10% of their time outdoors [47]. In spite of numerous reports of human thermal preferences inside buildings and codified climatic prescriptions (e.g. ASHRAE Standard 55) for construction of interior spaces, data on the climates actually achieved in houses, throughout the year, have not been widely reported.

We also identified outdoor climates from around the world that are most climatically similar (e.g. in terms of temperature and humidity, by season) to the indoor climate of the homes we studied. North American homes were most similar in climate to the outdoor conditions of west central Kenya (*C* = 2.938). The mean maximum temperature (average of all seasons) in the North American homes was 25.35°C compared with 25.06°C for the conditions outdoors in west central Kenya. The mean vapour pressure was 12.58 hPa for North American homes and was similar to the outdoor conditions in west central Kenya (12.96 hPa).

When humans adjust the climates within their homes, it is unlikely that most are consciously attempting to emulate the climatic conditions of some outdoor location in another country or continent. Instead, they are almost certainly attempting to achieve climatic conditions that result in thermal comfort. They do so to such an extent that indoor climate is no longer well correlated to outdoor climate (table 1). Based purely on its indoor temperature and humidity, you would be unlikely to discern whether a house from our dataset was in Wyoming or Mississippi. Of the two climatic variables, we considered, indoor humidity was more strongly correlated with outdoor conditions than was the case for temperature, but this correlation was weak. The extent to which humans have decoupled indoor and outdoor climate is likely to be the most extreme in nature. Even honeybee nests, for example, which are actively buffered from outdoor conditions, still vary in response to outdoor conditions.

In general, mammals, including humans, have evolved the ability to regulate their body temperatures via behaviour and autonomic responses. Human autonomic control has the capacity to maintain brain and core temperature over a range of environmental conditions [48]. Moreover, humans acclimate relatively quickly to new climatic conditions [49] and the evolution of hypothalamic controlled body temperatures, along with behavioural and cultural advances, may have allowed humans to expand the range of climatic conditions of their niche. So why do humans expend such extraordinary expense to maintain constant indoor climates [50] when such climates are not necessary for survival, especially given the plasticity of human temperature acclimation (e.g. *ama* divers to endurance athletes)? Probably, it is because these climates are comfortable.

In mammals, the perception of whether a climate is comfortable or not is an important driver of climate seeking behaviour [51], as a comfortable climate produces conditions that allow an individual to remain within their thermoneutral zone (TNZ). The TNZ is the range of environmental conditions where, for a given animal, heat loss equals gain and core body temperature is maintained [52]. When an individual is outside of this range of conditions, the individual may adjust climatic conditions behaviourally, physiologically or psychologically to adapt to the climatic conditions and ultimately perceive thermal comfort [51,53]. These TNZs are mutable and may change with an individual's climatic history or habituation of an indoor space (i.e. the Adaptive Comfort Model), but the methods to achieve thermal comfort remain the same [53,54]. Interestingly, the range of the mean indoor temperature recorded by citizen scientists in their homes and the 100 most climatically similar global grid cells (figure 3) largely fall within the TNZs (24–30°C) for primates including humans [23]. A comparison of climate-related physiological parameters between humans and a selection of non-human primates is included in table 3. We hypothesize that indoor climates largely correspond with our TNZ because our ancestors evolved thermal preferences that led them to favour (and ultimately build) these climates.

**Table 3.** Climate-related values for select primate species. Variables include animal husbandry recommendations for temperature (*T*_Husbandry_) and relative humidity (RH_Husbandry_), natural habitat temperature (*T*_Habitat_), normal adult body temperature (*T*_Body_) and thermoneutral zone (TNZ). *T*_Husbandry_ and RH_Husbandry_ values, [55]. *T*_Habitat_ values, Primate Info Network, Wisconsin National Primate Research Center, University of Wisconsin – Madison, accessed 10 April 2017; http://pin.primate.wisc.edu). *T*_Body_ and TNZ values, [23,56–60].

species	*T*_Husbandry_(°C)	RH_Husbandry_ (%)	*T*_Habitat_ (°C)	*T*_Body_ (°C)	TNZ (°C)
*Gorilla beringei*	18.3–29.4	30–70	3.9–14.5	unknown	unknown
*Gorilla gorilla*	18.3–29.4	30–70	23	35.5	unknown
*Homo sapiens*	n.a.	n.a.	n.a.	37	25–30
*Pan paniscus*	18–22	50–60	20–30	unknown	unknown
*Pan troglodytes*	15.6–29.4	30–70	18.5–30	37.25	17–29
*Pongo abelii*	18–28	30–70	17–34.2	unknown	unknown
*Pongo pygmaeus*	18–28	30–70	18–37.5	37	unknown

Perhaps not surprising, in light of the TNZ hypothesis, the temperature people prefer overlaps with much of the geographical area in which key events in hominid evolution and, for that matter, early civilization occurred [48]. We hypothesize that natural selection favoured human preferences and thermal traits that allowed human ancestors to live in those climates. However, as humans moved out of those environments they faced new climates. Strong evidence suggests that the selective pressures imparted by climate has altered human genomes [24,61,62]. In addition, new climates led to cultural responses such as the use of fire for heat [63], clothing [26] and shelter [64], all of which modified the climate to which individuals were exposed. We argue that modern temperatures in homes are a continuation of this same effort, but the technological ability of humans to modify climate has led to the extreme scenario, where fossil fuels are cheap, and (North American) indoor climates closely align with TNZs. Moreover, air-conditioned buildings with closed ventilation combined with changing indoor climatic expectations have also led to narrower ranges of human thermal comfort [30,53]. However, many questions remain. For example, do wealthy homeowners (or striving homeowners) keep their homes colder than is preferred in hot places to display wealth (and vice versa)? Do genetic backgrounds of homeowners influence preferred climates? How do these climates affect our health and well-being? For example, indoor climates are less variable [65] than outdoor climates and this reduced variability may lead to health issues such as obesity or diabetes [66,67].

Our results also offer a hypothesis about the likely origin of human home-associated species, as indoor climates probably favour certain lineages, those pre-adapted to indoor climates. We hypothesize that the assemblage of species that colonize our homes are likely to be those with thermal preferences/tolerances similar to us, which is to say species from relatively dry, relatively warm climates, including north and eastern Africa, but also much of the Middle East. Moreover, predictions can be made about the communities of home associates through time and space, as climate, home technologies and fortunes change. We know that climate preferences in homes differ among regions [25,29], and the USA is probably an extreme case, where indoor climates most reflect resource availability and culture, rather than economic and environmental costs.

Our characterization of the indoor climate of North American homes and the identification of the outdoor climates most similar to these homes opens a new line of inquiry. Why do we prefer these climatic conditions? Do the climates of modern houses reflect our ancestral climates? When and where did we evolve these modern climate preferences, and what are the contributions of genetic and cultural evolution to these preferences? Interestingly, the majority of 100 most climatically similar outdoor locations were located in the hot and seasonably dry northeastern Africa, a region rich in hominid fossils and evolution [68].

As a first step, we presented a simple comparison between the indoor climate of these North American homes and the climatic conditions experienced by some non-human primates (i.e. great apes). We found that climatic conditions generally overlapped. However, no *a priori* predictions seem to exist for which global climate we might favour in our homes, and future work should test the simplest one, namely that we tend to attempt to recreate the conditions from which we evolved, before we had the ability to make homes, the ones to which our physiologies are adapted.

## Supplementary Material

Indoor home climate data

## Supplementary Material

Appendix A

## Supplementary Material

Table S1

## Data accessibility

The datasets supporting this article have been uploaded as part of the supplementary material.
