# Supplementary material for: Human indoor climate preferences approximate specific geographies
Source: R Soc Open Sci. 2019 Mar 20;6(3):180695. doi: 10.1098/rsos.180695 (PMC6458351; doi:10.1098/rsos.180695)
Supplement: Appendix A [file rsos180695supp2.pdf]

Supplementary material for Just, Nichols, and Dunn – Indoor climates approximate specific geographies

Appendix A
Root mean square error (temperature, vapor pressure)

We calculated the root mean square errors between North American indoor and global outdoor climates, using the climatic parameters air temperature (°C) and vapor pressure (hPa), to determine if indoor climates approximated outdoor climates of specific geographies. To calculate the RMSE for temperature we used the following three temperature variables: minimum mean air temperature for winter, mean air temperature for spring/autumn, and maximum air temperature for summer. To calculate the RMSE for vapor pressure we used the following three vapor pressure variables: mean vapor pressure for (1) winter, (2) spring/autumn, and (3) summer. Seasons where defined as follows for the northern and southern hemispheres respectively: December – February (winter/summer), March - May (spring/autumn), June – August (summer/winter), September – November (autumn/spring). Spring and autumn were analyzed as one season, averaging spring and autumn values as needed.

We calculated the RMSE (described in Chai and Draxler (2014)) between each home and global grid cell, using the climate variables described above.

RMSE Results
Temperature (TMP)
The minimum RMSE TMP (2.31 °C) based on the mean indoor temperature of all USA study homes and outdoor global grid cells was found at 159.75 W, 22.25 N (Hawaii, USA)

The maximum RMSE TMP (52.60 °C) based on the mean indoor temperature of all USA study homes and outdoor global grid cells was found at 39.25 W, 79.75 N (Greenland)

Vapor pressure (VAP)
The minimum RMSE VAP (2.26 hPa) based on the mean indoor vapor pressure of all USA study homes and outdoor global grid cells was found at 19.75 E, 27.75 N (Libya)

The maximum RMSE VAP (20.31 hPa) based on the mean indoor vapor pressure of all USA study homes and outdoor global grid cells was found at 55.75 W, 4.25 S (Brazil)

Figures

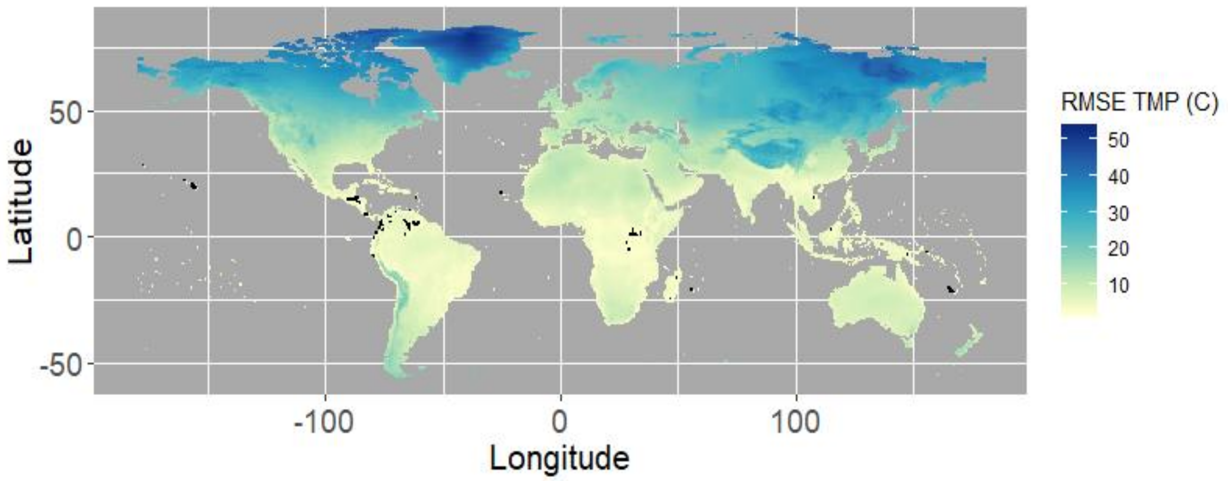

Fig. A1 Map depicting the root mean square error (RMSE) between the mean indoor temperature of the North American homes (*n* = 37; 2013-2014) and the outdoor climate of terrestrial 0.5° global grid cells (*n* = 67,420; 2012). Dissimilarity increases as *RMSE* increases. Cells depicted in black are those grid cells with the temperature conditions most similar to the average North American home (*n* = 100)

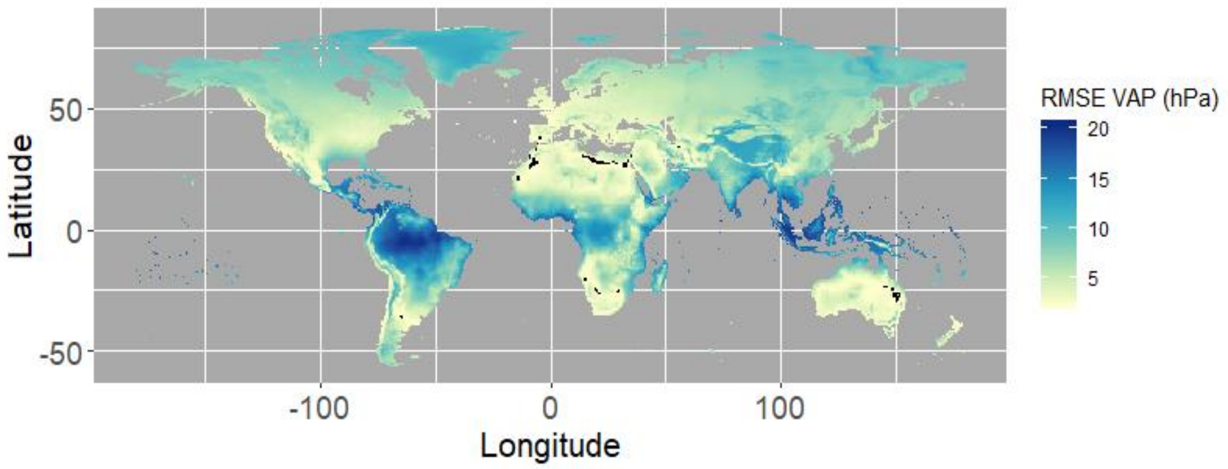

Fig. A2 Map depicting the root mean square error (RMSE) between the mean indoor vapor pressure of the North American homes (*n* = 37; 2013-2014) and the outdoor climate of terrestrial 0.5° global grid cells (*n* = 67,420; 2012). Dissimilarity increases as *RMSE* increases. Cells depicted in black are those grid cells with the vapor pressure conditions most similar to the average North American home (*n* = 100)

Tables

Table A1 Results of climate dissimilarity analysis between the indoor climate of a North American home (*n* = 37) and 67,420 global terrestrial grid cells. RMSE $TMP_{nearest}$ is the minimum value of the root mean square error for temperature (°C) for that state. The country where the center (latitude and longitude) of the grid cell is located is listed as the nearest country. RMSE $TMP_{Top\ 100}$ is the mean minimum value of RMSE TMP (standard error) for the 100 most climatically similar global grid cells for that state, the corresponding country represents the most frequently observed country from the 100 most similar grid cells based on smallest RMSE TMP.

State	RMSE $TMP_{nearest}$	Country (nearest)	Latitude	Longitude	RMSE $TMP_{Top\ 100}$	Country (Top 100)
Alabama	0.237	Equatorial Guinea	3.25	8.75	1.159 (0.19)	Cameroon
Alaska	0.275	United States of America	19.75	-155.75	1.034 (0.3)	Namibia
Arizona	1.725	Paraguay	-26.75	-56.25	2.023 (0.12)	Yemen
Arkansas	0.134	Colombia	6.25	-76.25	0.987 (0.35)	Colombia
California	0.401	Uganda	-0.75	30.25	0.972 (0.18)	Brazil
Connecticut	0.105	Democratic Republic of the Congo	-0.25	28.75	0.856 (0.19)	Madagascar
Delaware	0.324	Myanmar	21.25	99.75	0.825 (0.2)	Angola
Florida	0.496	Equatorial Guinea	3.25	8.75	1.104 (0.19)	Cameroon
Georgia	0.157	United States	20.75	-156.75	0.869 (0.28)	Colombia
Hawaii	0.231	Bahamas	20.75	-73.25	0.536 (0.11)	Cuba
Illinois	0.093	United States	18.75	-155.75	0.765 (0.21)	Colombia
Kansas	0.077	Indonesia	-3.75	126.25	0.317 (0.08)	Venezuela
Kentucky	0.521	Malaysia	4.25	115.75	1.013 (0.18)	Indonesia
Louisiana	0.642	United Republic of Tanzania	-4.75	38.75	0.895 (0.09)	Brazil

Maryland	1.453	Sao Tome and Principe	0.25	6.75	2.709 (0.23)	Cameroon
Massachusetts	0.295	Laos	22.25	101.75	0.572 (0.11)	Brazil
Michigan	1.354	Uganda	-0.25	32.25	1.757 (0.14)	United Republic of Tanzania
Minnesota	2.299	Ecuador	-0.75	-91.25	2.627 (0.11)	Democratic Republic of the Congo
Missouri	0.745	Sao Tome and Principe	0.25	6.75	1.825 (0.23)	Cameroon
Nebraska	0.136	Peru	-5.75	-77.25	0.453 (0.12)	United Republic of Tanzania
Nevada	0.036	South Sudan	4.25	32.75	0.518 (0.11)	Democratic Republic of the Congo
New Hampshire	0.563	Democratic Republic of the Congo	-0.75	28.75	1.083 (0.14)	Peru
New Mexico	0.140	Cape Verde	16.75	-24.75	0.539 (0.14)	Venezuela
North Carolina	0.162	Burundi	-4.25	29.75	0.588 (0.14)	Madagascar
North Dakota	0.365	French Polynesia	-17.75	-149.25	1.027 (0.22)	Colombia
Oklahoma	0.731	Colombia	7.25	-75.25	1.09 (0.15)	Indonesia
Oregon	0.147	Namibia	-20.25	13.75	0.782 (0.22)	Namibia
South Carolina	0.921	Equatorial Guinea	3.25	8.75	1.591 (0.21)	Cameroon
South Dakota	0.456	Colombia	3.75	-74.75	1.097 (0.24)	Colombia
Tennessee	0.161	United Republic of Tanzania	-3.25	34.75	0.529 (0.12)	United Republic of Tanzania
Utah	0.227	Pitcairn	-25.25	-130.25	0.851 (0.28)	Colombia
Vermont	0.424	Kenya	-1.25	35.25	0.894 (0.16)	Brazil
Virginia	0.127	Democratic Republic of the Congo	1.75	30.75	0.83 (0.27)	Colombia

Washington	0.317	Colombia	3.75	-74.75	1.124 (0.25)	Colombia
West Virginia	0.090	Kenya	0.25	34.75	0.488 (0.12)	Angola
Wisconsin	0.165	Colombia	1.75	-75.75	0.836 (0.29)	Colombia
Wyoming	1.534	Ecuador	-0.75	-91.25	1.87 (0.1)	Democratic Republic of the Congo

Table A2 Results of climate dissimilarity analysis between the indoor climate of a North American home (*n* = 37) and 67,420 global terrestrial grid cells. RMSE $VAP_{nearest}$ is the minimum value of the root mean square error for vapor pressure (hPa) for that state. The country where the center (latitude and longitude) of the grid cell is located is listed as the nearest country. RMSE $VAP_{Top\ 100}$ is the mean minimum value of RMSE VAP (standard error) for the 100 most climatically similar global grid cells for that state, the corresponding country represents the most frequently observed country from the 100 most similar grid cells based on smallest RMSE VAP.

State	RMSE $VAP_{nearest}$	Country (nearest)	Latitude	Longitude	RMSE $VAP_{Top\ 100}$	Country (Top 100)
Alabama	0.221	Peru	-14.25	-75.25	0.575 (0.13)	Australia
Alaska	0.086	Australia	-31.25	132.25	0.187 (0.05)	Australia
Arizona	0.046	United Kingdom	56.75	-5.75	0.354 (0.09)	United Kingdom
Arkansas	0.195	Mexico	17.75	-96.75	0.494 (0.12)	Ethiopia
California	0.017	Australia	-26.75	116.25	0.315 (0.08)	Australia
Connecticut	0.022	China	26.25	104.75	0.267 (0.08)	Russia
Delaware	0.081	Mauritania	25.25	-8.25	0.237 (0.06)	Australia
Florida	0.286	Papua New Guinea	-6.25	145.25	0.836 (0.2)	Saudi Arabia
Georgia	0.220	Angola	-14.25	15.75	0.657 (0.16)	Angola
Hawaii	0.243	Nicaragua	13.75	-84.75	0.548 (0.11)	Democratic Republic of the Congo
Illinois	0.306	Libya	30.25	10.25	0.48 (0.05)	Algeria

Kansas	0.123	Western Sahara	22.75	-13.25	0.435 (0.1)	Egypt
Kentucky	0.255	Bolivia	-17.25	-65.25	0.562 (0.08)	Argentina
Louisiana	0.071	Ethiopia	8.25	42.75	0.517 (0.18)	Saudi Arabia
Maryland	0.019	Mauritania	24.25	-8.25	0.245 (0.07)	Australia
Massachusetts	0.076	Egypt	27.25	30.25	0.225 (0.07)	Australia
Michigan	0.030	Egypt	27.25	30.75	0.235 (0.07)	Australia
Minnesota	0.159	Mexico	30.75	-112.75	0.572 (0.1)	Australia
Missouri	0.477	Libya	30.75	10.75	0.66 (0.07)	Algeria
Nebraska	0.054	South Africa	-27.25	26.75	0.243 (0.07)	Australia
Nevada	0.037	Argentina	-39.25	-68.75	0.293 (0.09)	Argentina
New Hampshire	0.117	Croatia	44.75	14.75	0.325 (0.08)	United States of America
New Mexico	0.111	Spain	41.25	-0.25	0.285 (0.06)	Australia
North Carolina	0.284	Angola	-16.75	17.25	0.621 (0.09)	Greece
North Dakota	0.181	Turkmenistan	39.25	55.75	0.295 (0.04)	Algeria
Oklahoma	0.284	Mexico	18.75	-99.75	0.581 (0.09)	Argentina
Oregon	0.091	Colombia	10.75	-73.75	0.699 (0.21)	Saudi Arabia
South Carolina	0.351	Angola	-10.75	15.75	0.925 (0.16)	Ethiopia
South Dakota	0.133	Bolivia	-16.75	-67.25	0.33 (0.06)	Libya
Tennessee	0.352	Bolivia	-17.75	-64.75	0.568 (0.08)	Libya
Utah	0.089	Saudi Arabia	27.75	46.75	0.491 (0.14)	Iraq
Vermont	0.099	United States of America	32.75	-102.75	0.311 (0.09)	United States of America
Virginia	0.060	Namibia	-22.25	19.75	0.305 (0.07)	Australia

Washington	0.080	Saudi Arabia	24.25	48.25	0.487 (0.14)	Australia
West Virginia	0.150	Chad	15.75	16.25	0.389 (0.09)	Chad
Wisconsin	0.126	Albania	41.75	19.75	0.427 (0.1)	Turkey
Wyoming	0.072	Spain	40.75	-4.75	0.278 (0.08)	Libya

Chai, T., & Draxler, R. R. (2014). Root mean square error (RMSE) or mean absolute error (MAE)? -Arguments against avoiding RMSE in the literature. *Geoscientific Model Development*, *7*(3), 1247–1250. https://doi.org/10.5194/gmd-7-1247-2014
